# Supplementary material for: Aryl Hydrocarbon Receptor Activation Downregulates IL-33 Expression in Keratinocytes via Ovo-Like 1
Source: J Clin Med. 2020 Mar 24;9(3):891. doi: 10.3390/jcm9030891 (PMC7141508; doi:10.3390/jcm9030891)
Supplement: Supplementary file 1 [file jcm-09-00891-s001.pdf]

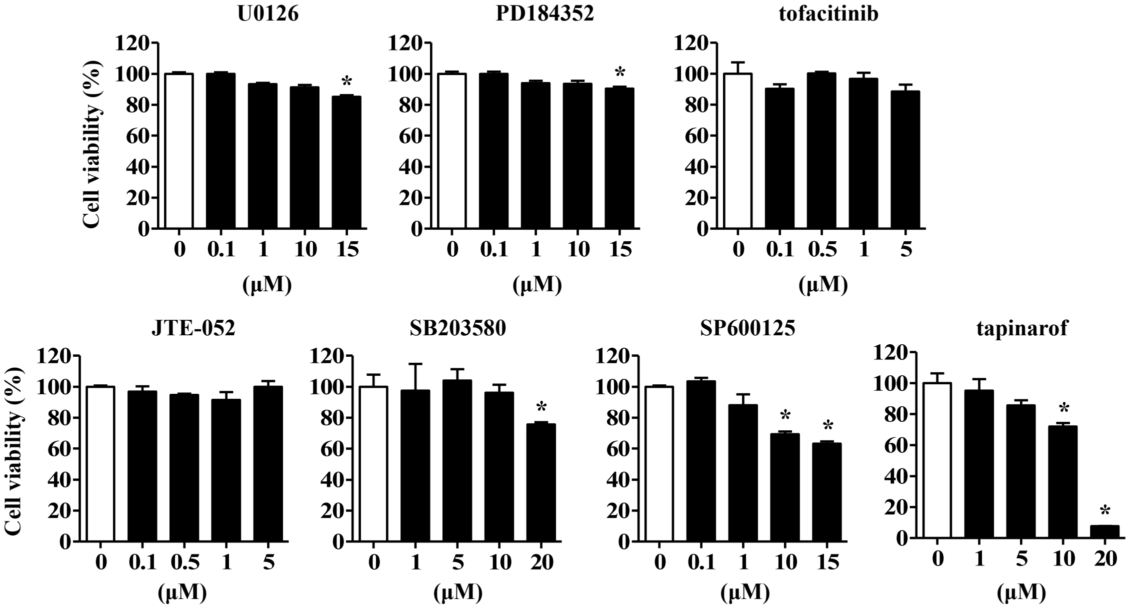

Supplementary Figure S1. Cell viability. NHEKs were treated with U0126, PD184352, tofacitinib, JTE-052, SB203580, SP600125, and tapinarof at the indicated doses for 24 h. WST-1 cell proliferation assay was performed to evaluate cell viability. Statistically significant differences between the expression of control and NHEKs treated with U0126, PD184352, SB203580, SP600125, or tapinarof are presented: *P < 0.05.

Primers for qRT-PCR

Gene	Forward primer	Reverse primer
IL33	5’-AGCCTTGTGTTTCAAGCTGGG-3’	5’-TTGTGCTTTCTACCTGTTTTCAGTG-3’
CCL26	5’-GACCTGGGTGCGAAGCTATG-3’	5’-TCCAAGCGTCCTCGGATGAA-3’
AHR	5’-ATCACCTACGCCAGTCGCAAG-3’	5’-AGGCTAGCCAAACGGTCCAAC-3’
OVOL1	5’-GATCTACGTGCCAGTCAGCC-3’	5’-GCTCATGTTCAAAGCCAGCG-3’
β-actin	5’-ATTGCCGACAGGATGCAGA-3’	5’-GAGTACTTGCGCTCAGGAGGA-3’

Supplementary Figure S2. Primers for qRT-PCR.

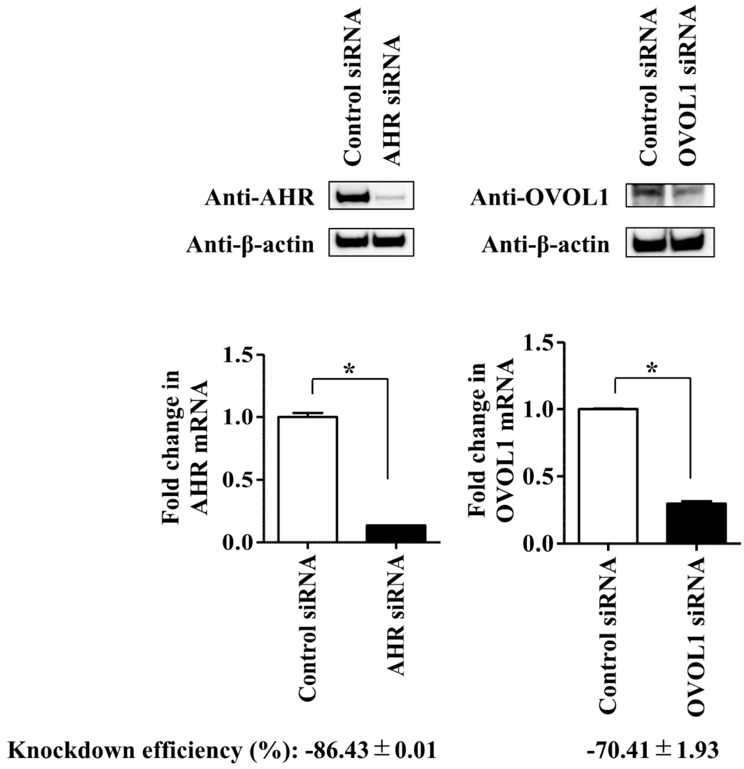

Supplementary Figure S3. siRNA transfection against either AHR or OVOL1 downregulated mRNA and protein expression of either AHR or OVOL1 in NHEKs. NHEKs were transfected with either control siRNA (si-control), siRNA against AHR (si-AHR), or siRNA against OVOL1 (si-OVOL1). (Upper lane) AHR or OVOL1 protein expression was analyzed by Western blotting with an anti-AHR antibody or an anti-OVOL1 antibody. The data are representative of experiments repeated three times with similar results. (Lower lane) AHR or OVOL1 mRNA expression was analyzed by qRT-PCR. Data are expressed as mean ± S.E.M.; n = 3 for each group. *$P < 0.05$.

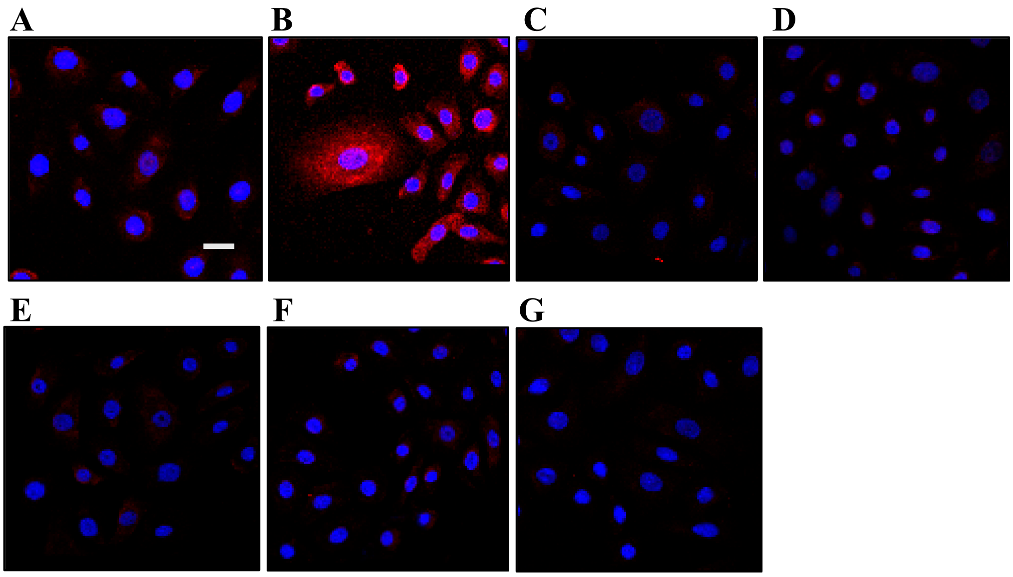

Supplementary Figure S4. Immunofluorescence staining using anti-human CCL26 antibody. NHEKs treated with PBS (A), IL-4 (10 ng/ml) (B), tofacitinib (500 nM) (C), tofacitinib (500 nM) plus IL-4 (10 ng/ml) (D), JTE-052 (1000 nM) (E) and JTE-052 (1000 nM) plus IL-4 (10 ng/ml) (F) for 24 h. (G) Isotype negative control. The scale bar represents 25 μm. The data are representative of experiments repeated three times with similar results.

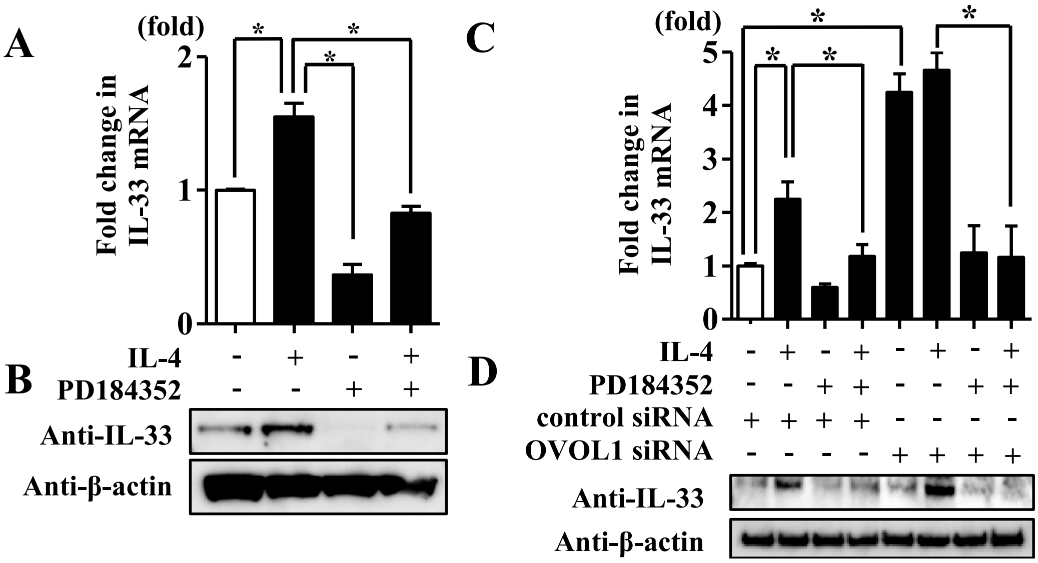

Supplementary Figure S5. PD184352, a MEK inhibitor, inhibited upregulation of IL-33 expression induced by IL-4 in NHEKs and OVOL1-knockdowned NHEKs. (A and B) NHEKs were treated with IL-4 (10 ng/ml) in the presence or absence of PD184352 (10 μM) for 24 h. (C and D) NHEKs were transfected with control siRNA (si-control) and siRNA against OVOL1 (si-OVOL1) and subsequently treated with IL-4 (10 ng/ml) in the presence or absence of PD184352 (10 μM) for 24 h. (A and C) IL-33 expression was analyzed by qRT-PCR. Data are expressed as mean ± S.E.M.; n = 3 for each group. *P < 0.05. (B and D) IL-33 expression was analyzed by Western blotting with an anti-IL-33 antibody. The data are representative of experiments repeated three times with similar results.

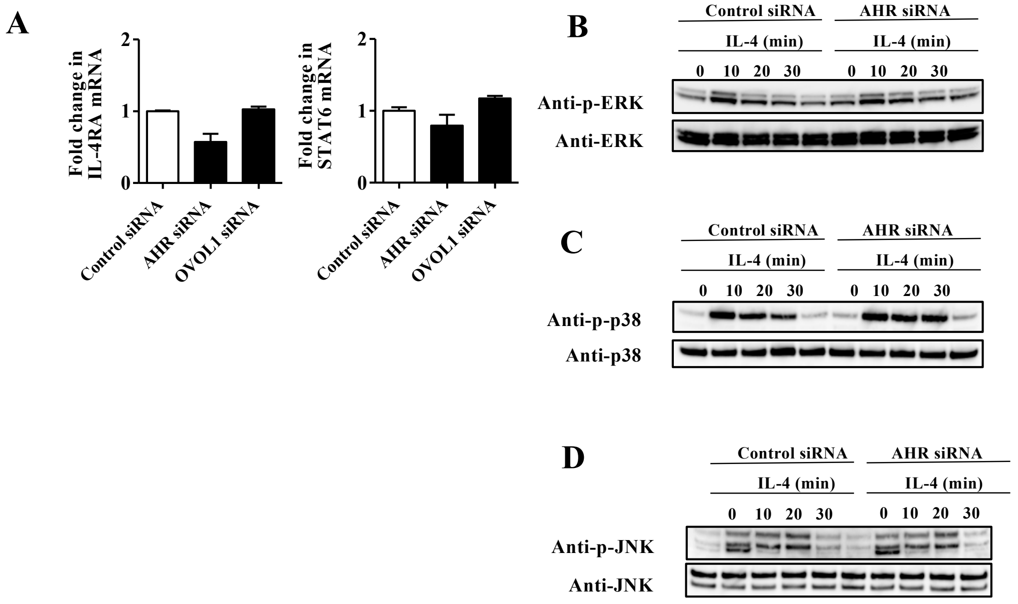

Supplementary Figure S6. Knockdown of either AHR or OVOL1 did not alter mRNA expression of IL-4RA and STAT6 in NHEKs. (A) NHEKs were transfected with either control siRNA (si-control), siRNA against AHR (si-AHR), or siRNA against OVOL1 (si-OVOL1) and subsequently analyzed mRNA expression of IL-4RA and STAT6. Data are expressed as mean ± S.E.M.; n = 3 for each group. Primer sequence: *IL-4RA* forward primer 5′-GAGTGAAAACGACCCGGCAG-3′, reverse primer 5′-TCCCTGTAGGAGTTGTGCCA-3′. *STAT6* forward primer 5′-CACCGTTTGAGGAGAGCCTG-3′, reverse primer 5′-CTCCACCAGGAAGCAACTGG-3′ (B) NHEKs transfected with either si-control or si-AHR were treated with IL-4 (10 ng/ml) for the indicated period and subjected to Western blotting analysis with an anti-phosphorylated ERK-1/2 and anti-ERK-1/2 antibody (B), anti-phosphorylated p-

38 and anti-p38 antibody (C), or anti-phosphorylated JNK antibody and anti-JNK antibody (D). The data are representative of experiments repeated three times with similar results.

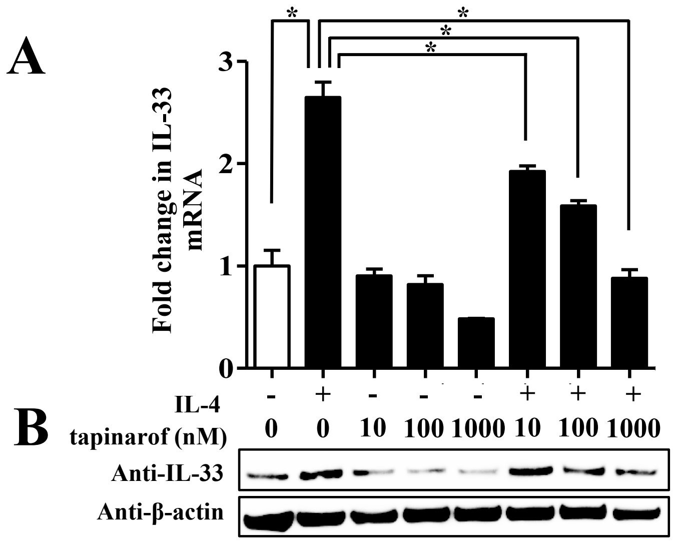

Supplementary Figure S7. Tapinarof, an AHR agonist, inhibited IL-4-induced upregulation of IL-33 expression in NHEKs. (A and B) NHEKs were treated with IL-4 (10 ng/ml) for 24 h in the presence or absence of tapinarof (10, 100, and 1000 nM). (A) IL-33 expression was analyzed by qRT-PCR. Data are expressed as mean ± S.E.M.; n = 3 for each group; *P < 0.05. (B) IL-33 expression was analyzed by Western blotting with an anti-IL-33 antibody. The data are representative of experiments repeated three times with similar results.
